# Human Mesenchymal Stem Cell-Treated Regulatory CD23^+^CD43^+^ B Cells Alleviate Intestinal Inflammation

**DOI:** 10.7150/thno.32260

**Published:** 2019-06-24

**Authors:** Xiaoyong Chen, Chuang Cai, Dijing Xu, Qiuli Liu, Shuwei Zheng, Longshan Liu, Gang Li, Xiaoran Zhang, Xiaoping Li, Yuanchen Ma, Li Huang, Jieying Chen, Jiahao Shi, Xin Du, Wenjie Xia, Andy Peng Xiang, Yanwen Peng

**Affiliations:** 1 The Biotherapy Center, the Third Affiliated Hospital, Sun Yat-sen University, Guangzhou, 510630, China.; 2 Center for Stem Cell Biology and Tissue Engineering, Key Laboratory for Stem Cells and Tissue Engineering, Ministry of Education, Sun Yat-sen University, Guangzhou, 510080, China.; 3 Department of Pathophysiology, Zhongshan School of Medicine, Sun Yat-sen University, Guangzhou, 510080, China.; 4 Organ Transplant Center, the First Affiliated Hospital, Sun Yat-sen University, Guangzhou, 510080, China.; 5 Hematological Department of Guangdong Province People's Hospital, Guangzhou 510080, China.; 6 Institute of Blood Transfusion, Guangzhou Blood Centre, Guangzhou, 510095, China; 7 Department of Biochemistry, Zhongshan School of Medicine, Sun Yat-sen University, Guangzhou, 510080, China.; 8 Key Laboratory of Protein Modification and Degradation, School of Basic Medical Sciences, Affiliated Cancer Hospital and Institute of Guangzhou Medical University, Guangzhou 511436, China

**Keywords:** mesenchymal stem cells, regulatory B cells, inflammatory bowel disease, interleukin-10

## Abstract

**Rationale:** Mesenchymal stem cells (MSCs) have been demonstrated to ameliorate inflammatory bowel disease by their actions on multiple immune cells, especially on regulatory B cells (Breg cells). However, the phenotypes and functions of human MSCs (hMSCs)-treated Breg cell subsets are not yet clear.

**Methods:** Purified B cells were cocultured with MSCs and the phenotypes and immunomodulatory functions of the B cells were analyzed by FACS and proliferation assays *in vitro*. Also, a trinitrobenzenesulfonic acid-induced mouse colitis model was employed to detect the function of MSC-treated Breg cells *in vivo*.

**Results:** We demonstrated that coculturing with hMSCs significantly enhanced the immunomodulatory activity of B cells by up-regulating IL-10 expression. We then identified that a novel regulatory B cell population characterized by CD23 and CD43 phenotypic markers could be induced by hMSCs. The CD23^+^CD43^+^ Breg cells substantially inhibited the inflammatory cytokine secretion and proliferation of T cells through an IL-10-dependent pathway. More significantly, intraperitoneal injection of hMSCs ameliorated the clinical and histopathological severity in the mouse experimental colitis model, accompanied by an increase in the number of CD23^+^CD43^+^ Breg cells. The adoptive transfer of CD23^+^CD43^+^ B cells effectively alleviated murine colitis, as compared with the CD23^-^CD43^-^ B cells. Treatment with CD23^+^CD43^+^ B cells, and not hMSCs, substantially improved the symptoms of colitis in B cell-depleted mice.

**Conclusion:** the novel CD23^+^CD43^+^ Breg cell subset appears to be involved in the immunomodulatory function of hMSCs and sheds new light on elucidating the therapeutic mechanism of hMSCs for the treatment of inflammation-related diseases.

## Introduction

Inflammatory bowel disease (IBD), a chronic autoimmune intestinal disease, is associated with multiple pathogenic factors including environmental changes, an array of susceptibility gene variants, qualitatively and quantitatively abnormal gut microbiota, and a broadly dysregulated immune response 1. Although there have been significant advances in the medical treatment of this disease, many patients become refractory to the available therapeutic options. Mesenchymal stem cells (MSCs), with the immunomodulatory capability to downregulate immune reactivity and promote tissue healing 2, are becoming a promising therapeutic option for severe refractory cases, especially when surgery is not feasible 3.

MSCs are multipotent progenitor cells that are derived from bone marrow, adipose tissue, umbilical cord, and other tissues 4 and differentiate into various mesodermal lineages under relevant induction conditions 4, 5. MSCs have been shown to modulate a wide range of immune cells, inhibit the proliferation and function of T cells and NK cells 6-8, down-regulate maturation and function of dendritic cells 9, 10, suppress the proliferation of activated B cells, and reduce antibody production by B cells 11, 12 indicating that MSCs may be a novel candidate for treating a variety of inflammatory diseases.

B cells have long been reported to possess immunosuppressive ability, ever since they were first shown to inhibit delayed-type hypersensitivity skin reactions 13. Since then, tremendous advances have been made in characterizing the functional mechanisms of Breg cells. This has led to the identification of a novel subset of regulatory B cells (Breg) cells known as B10 cells, which regulate immune responses by producing the anti-inflammatory cytokine, interleukin-10 (IL-10) 14. They have been shown to exert immunoregulation by inhibiting T cell responses (Th1, Th2, and Th17) 15-17, maintaining the pool of regulatory T cells (Tregs), and converting effector T cells into Foxp3^-^IL-10^+^CD4^+^ regulatory T cells 16, 18. Growing evidence suggests that B10 cells act as potent regulators of allergic and autoimmune diseases, infection, cancer, and rejection after transplantation 14. We recently showed that MSCs increased the frequency of B10 cells *in vitro* and *in vivo*, and alleviated experimental colitis associated with the increase of Breg cells 19, 20. However, B10 cells are not limited to any particular phenotypic subset and the phenotype and characteristics of these MSCs- treated Breg cells still need to be elucidated.

To date, multiple Breg cell subsets with common phenotypic characteristics have been described. In mice, a B10 subset with a phenotype of CD1d^hi^CD5^+^CD19^+^ was demonstrated to represent Breg cells 21. However, the cell surface markers of this CD1d^hi^CD5^+^ Breg subset were shared with a variety of other B cell subsets, including CD5^+^ B-1a B cells 22, CD1d^hi^CD23^-^IgM^hi^CD21^+^ marginal zone B cells 23, and CD1d^hi^CD21^+^CD23^+^IgM^hi^ T2 marginal zone precursor (T2-MZP) B cells 24, all of which contain B10 cells. Likewise, human Breg cells were predominantly identified by their expression of IL-10, which was enriched in CD24^hi^CD38^hi^ transitional B cells 25, CD24^hi^CD27^+^ memory B cells 26, and CD27^hi^CD38^hi^ plasmablast B cells 27. Furthermore, a previous study showed that human CD25^hi^CD86^hi^CD1d^hi^ B cells were also IL-10-producing Breg cells 28. Thus, Breg cells are believed to be heterogeneous cells whose subsets express specific candidate markers that may be altered under different immune responses. This could explain some of the discrepancies in the definition of different Breg cell subsets across various experimental settings. It is not yet known, however, whether the observed differences were due to the existence of distinct Breg cell population or to changes related to the immunological environment 29.

Therefore, the Breg cells that are regulated by MSCs may differ from other subsets and may possess specific phenotypic markers. The characteristics of these MSC-treated Breg cells have not yet been fully elucidated. Here, we investigated the characteristics of MSC-treated Breg cells and their potential role in the MSC-based treatment of IBD.

## Materials and Methods

### Isolation and characterization of hMSCs

Heparin-treated bone marrow was obtained by iliac crest aspiration from healthy donors following the Declaration of Helsinki protocols with informed consent, and the protocol was approved by the relevant Ethics Review Board prior to initiation. In brief, marrow mononuclear cells were separated by Ficoll-Paque (1.077 g/mL; Amersham Biosciences, Uppsala, Sweden) density gradient centrifugation and seeded at a density of 1x10^5^/cm^2^ into T75 cell culture flasks. When cells grew to 80% confluence, they were detached by trypsin-EDTA and designated as passage 1. These cells were further passaged at a ratio of 1:3. As shown in **Figure S1**, these cells expressed the surface markers CD29, CD44, CD73, CD90, CD105, and CD166, but not the hematopoietic markers CD45 and CD34. At the 5th passage, the multipotent differentiation capacity of hMSCs was confirmed by the induction of adipogenic, osteogenic, and chondrogenic phenotypes, as previously described 19, 30, 31. These well-characterized 6th passage hMSCs were used in both *in vitro* and *in vivo* studies.

### Flow cytometry and cell sorting

The antibodies used in this study included anti-human IgD PerCp-Cy5.5, anti-human IgD PE- Cy7, anti-human IgM-APC, anti-human CD1d-PE, anti-human CD3-FITC, anti-human CD3-Horizon V450, anti-human CD4- Horizon V500, anti-human CD8-APC, anti-human CD10-PE, anti-human CD19- FITC, anti-human CD19-Horizon V500, anti-human CD21-PE-Cy7; anti-human CD23-PerCP-Cy5.5, anti- human CD23-PE, anti-human CD24-PE, anti-human CD25-PerCP-Cy5.5, anti-human CD25-APC-CY7, anti-human CD27-FITC, anti-human CD27-APC, anti- human CD38-PE-Cy7, anti-human CD38-PE, anti- human CD40-PE-Cy7, anti-human CD43-APC, anti- human CD43-FITC, anti-human IL-10-PE, anti-human IL-10-APC, anti-human IL10-AF488, anti-human IFN- r-APC, anti-human IFN-r-PE-CY7, anti-human TNF- a-PE, anti-human TNF-a-PerCP-Cy5.5, anti-mouse CD19-APC, anti-mouse CD19-PE-Cy7, anti-mouse CD23-FITC, anti-mouse CD43-PE, and anti-mouse IL-10-APC. All antibodies were from BD Bioscience. Fluorochrome-matched isotype controls were included. Samples were analyzed using a Gallios (Beckman Coulter) or a Cytoflex (Beckman Coulter) flow cytometer. Data were analyzed using the Flow Jo 10 software and Kaluza, and the results were reported as the percentage of positive cells or as mean fluorescence intensity compared to the isotype control.

CD3^+^CD4^+^CD25^-^ T cells, CD19^+^ B cells, CD19^+^ CD23^+^CD43^+^ B cells and CD19^+^CD23^-^CD43^-^ B cells were purified from PBMCs of healthy donors by FACS sorting. For adoptive transfer therapy, CD19^+^CD23^+^CD43^+^ B cells and CD19^+^CD23^-^CD43^-^ B cells were sorted from peritoneal cavity cells of mice.

For MSC-mediated CD23^+^CD43^+^ B cells sorting, the purified CD19^+^ B cells were cocultured with hMSCs in the presence of 4 μg/ml CpG ODN 2006 (InvivoGen) and 1 μg/ml trimeric CD40L (R&D Systems) for 2 days. Then, the B cells were harvested and sorted after incubating with antibodies against CD19, CD23 and CD43. Cells were sorted by BD influx or MoFlo Astrios EQ.

### Intracellular cytokine staining (ICCS)

For analysis of intracellular cytokine production, cells were stimulated with 0.2 μg/ml anti-CD3 mAb (BD Biosciences Pharmingen) for T cells, or 4 μg/ml CpG ODN 2006 (InvivoGen) and 1 μg/ml trimeric CD40L (R&D Systems) for B cells. BFA (10 μg/ml; Sigma), PMA (50 ng/ml; Sigma), and ionomycin (1 μg/ml; Sigma) were added for the last 6 hours. The cells were then incubated with antibodies against CD3 and CD19. The cells were washed, fixed, permeabilized, and intracellular cytokines were detected with anti-human IFN-γ-APC, anti-TNF-α-PE or anti-human IL-10-PE according to the manufacturer's instructions. Blocking experiments were performed with 10 μg/ml anti-IL-10 mAb (R&D Systems).

For analysis of B10 cells, the peritoneal cavity cells were harvested from mice and were incubated with BFA (10 μg/ml), PMA (50 ng/ml), and ionomycin (1 μg/ml) for 6 hours. The cells were stained with anti-mouse CD19 antibody, anti-mouse CD23 antibody and anti-mouse CD43 antibody, followed by anti-mouse IL-10 antibody intracellular staining according to the manufacturer's instructions.

### Proliferation assay

Purified CD19^+^ B cells were activated by CpG ODN 2006 (4 μg/ml) and CD40L (1 μg/ml) in the presence or absence of hMSCs for 48 hours, and then separately cocultured with T cells. CD3^+^CD4^+^CD25^-^ T cells were isolated, labeled with 5 μM CFSE (CellTrace™ CFSE Cell Proliferation kit; Invitrogen), and subjected to the following cultures: alone; with activated B cells at ratios of 10:1, 5:1, 2:1, and 1:1; or with activated hMSCs-treated B cells at ratios of 10:1, 5:1, 2:1, and 1:1. All T cells were stimulated with anti-CD3 mAb (0.2 μg/ml) and anti-CD28 mAb (1 μg/ml; BD Biosciences Pharmingen) for 96 hours. T cell proliferation was evaluated by flow cytometric analysis of CFSE dilution. Blocking experiments were performed with 10 μg/ml anti-IL-10 mAb (R&D Systems).

For the proliferation assay, purified CD23^+^CD43^+^ and CD23^-^CD43^-^ B cells were cultured with or without hMSCs in the presence CpG ODN 2006 (4 μg/ml) , CD40L (1μg/ml), and EdU (10μM, Invitrogen) for 96 hours. Cells were stained with the Click-iT® EdU Alexa Fluor® 488 Cell Proliferation Assay Kit (Invitrogen) according to the manufacturer's instructions. B cell proliferation was evaluated by flow cytometric analysis of EdU^+^ B cells.

### B cells/hMSCs culture assay

Purified CD19^+^ B cells were stimulated with 4 μg/ml CpG ODN 2006 and 1 μg/ml CD40L in the presence or absence of hMSCs, and the frequencies of IL-10 producing B cells or CD23^+^CD43^+^ B cells were evaluated. For CD23^-^CD43^-^ B cell conversion experiments, purified CD19^+^CD23^-^CD43^-^ B cells were stimulated with 4 μg/ml CpG ODN 2006 and 1 μg/ml CD40L in the presence or absence of hMSCs, and the increase in CD23^+^CD43^+^ B cells was evaluated. To explore whether the cell-cell contacts were involved in this process, B cells and hMSCs were cocultured separately using the Transwell assay. To explore which factor participated in this process, 1 μM indomethacin (a non-specific inhibitor for COX-2/ PGE2, Sigma), 1 μM NS398 (a specific inhibitor for COX-2/PGE2, Sigma), 1 mM 1-MT (a specific inhibitor for IDO, Sigma), 10ug/ml anti-HGF antibody (R&D Systems), 10ug/ml anti-IL-6 antibody (R&D Systems), 10ug/ml anti-IL-10 antibody (R&D Systems), and 1 mM L-NAME (a nonselective inhibitor of NOS, including human eNOS and murine iNOS; purchased from Beyotime Biotechnology) were added to the B/hMSCs cocultures.

### TNBS-induced colitis

Food (but not water) was withdrawn from BALB/c mice (5 per group) for 24 h, the mice were anesthetized with isoflurane, and a 3.5-French catheter was inserted into the colon 4 cm proximal to the anus. Subsequently, trinitrobenzenesulfonic acid (TNBS; 1.5 mg in 150 µL 50% ethanol; Sigma) was delivered to the lumen using a 1-mL syringe. The mice were maintained in a vertical position for 30 s to ensure the proper distribution of TNBS within the colon. Control mice received 50 µl of a 0.9% NaCl:Ethanol (50:50, v/v) solution. On the day after the above treatment, 1×10^6^ hMSCs were delivered by intraperitoneal injection.

All animal studies were carried out in accordance with the guidelines of the Sun Yat-sen University Institutional Animal Care and Use Committee.

### Colitis assessment

Body weight was monitored daily, and mice were euthanized on day 3 after the induction of colitis (the peak of the disease). Colons were collected without the cecum, measured for length, and evaluated for macro- or microscopic damage. Disease activity and scores were evaluated as previously described 20. The severity of colitis was assessed according to the colitis score, macroscopic damage, colon length and histological damage.

### Breg adoptive transfer

Mouse peritoneal cavity leukocytes were isolated following a previously published protocol 32. Briefly, 4 ml of RPMI 1640 medium was injected into the peritoneal cavity of sacrificed mice followed by gentle massage of the abdomen and recovery of the fluid with a large-gauge needle. CD19^+^CD23^+^CD43^+^ B cells and CD19^+^CD23^-^CD43^-^ B cells were sorted from the harvested peritoneal cavity leukocytes by FACS sorting. Subsequently, 1×10^6^ CD19^+^CD23^+^CD43^+^ B cells or CD19^+^CD23^-^CD43^-^ B cells were transferred intraperitoneally into recipient mice on day 1 after colitis induction.

### B cell depletion in mice

Mice were treated with the anti-mouse CD20 antibody according to the manufacturer's recommended procedures (BioLegend). In brief, mice were injected i.v. with 250 µg of anti-mouse CD20 antibody. The peripheral blood sample and peritoneal cells were collected before treatments and 7 days after antibody administration. Cells were then isolated and stained with anti-mouse CD19 antibody. The CD19^+^ B cells were detected by flow cytometry.

### RNA-Seq Data Process

Raw RNA-Seq data were processed using CLC workbench. GRCh37.p13 genome file was used. Gene referenced file came from NCBI. Transcripts per million normalized counts were used for the downstream analysis. PCA, hierarchical cluster, etc., were performed by local R scripts based on ggplot2, tidyr, etc.

### Statistical analysis

All data were expressed as mean ± SD. Statistical analyses were performed with the Prism5 Graphpad software (GraphPad Software, CA) using unpaired t-tests and two-way ANOVA. Statistical significance was set at p< 0.05.

## Results

### hMSCs enhance the immuno-modulatory function of B cells

**Figure 1:**
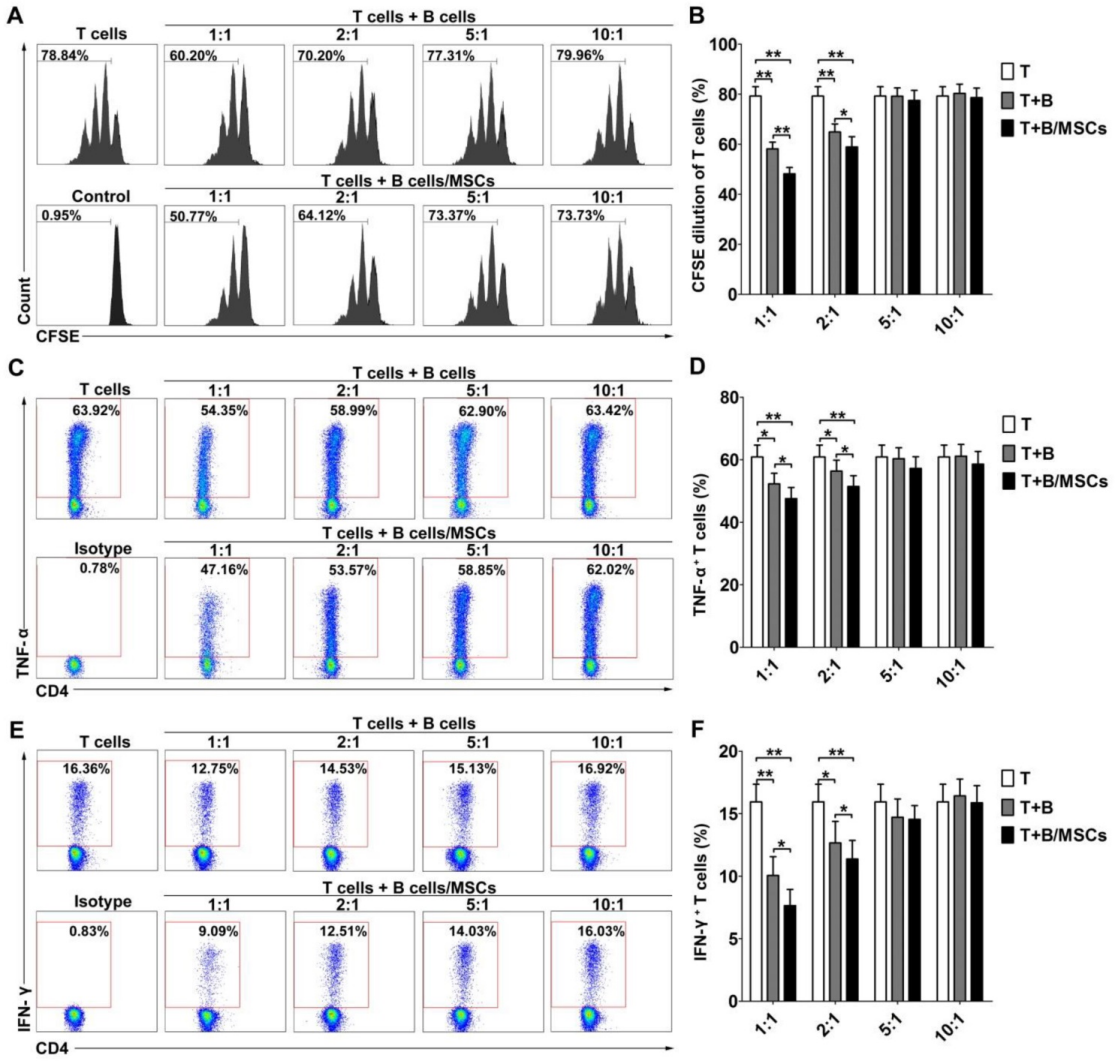
** hMSCs enhanced the immunosuppressive effect of B cells on T cell responses.** CpG ODN 2006 and CD40L-stimulated B cells or hMSCs-treated B cells were cocultured with sorted CD4^+^ T cells, and the proliferation and pro-inflammatory cytokine secretion of T cells were evaluated by flow cytometry. (A) Representative histograms of CFSE-labeled T cell proliferation at different T/B ratios. (B) Quantification of CFSE-diluted T cells. (C) Representative plots of TNF-α production by T cells at different T/B ratio. (D) Quantification of TNF-α-producing T cells. (E) Representative plots of IFN-γ production by T cells in different T/B ratio. (F) Quantification of IFN-γ-producing T cells. Data represent mean values ± SD of four independent experiments. *p<0.05; **p<0.01.

To investigate the effects of hMSCs on the regulatory functions of human B cells, we examined the inhibitory abilities of B cells and hMSC-treated B cells. As shown in **Figure S2**, CFSE-labeled CD4^+^ CD25^-^ T cells proliferated well over time in the presence of anti-CD3 mAb and anti-CD28 mAb. After 48h of stimulation by CpG and CD40L, activated B cells could inhibit T cell proliferation at low T/B ratio (**Figure 1A-B; *p*<0.01**). hMSC-treated B cells showed an even stronger suppressive effect at the same ratio (**Figure 1A-B; *p*<0.01 or 0.05**). Simultaneously, the activated B cells dose-dependently down-regulated the production of the inflammatory cytokines, TNF-α (**Figure 1C-D; *p*<0.01 or 0.05**) and IFN-γ (**Figure 1E-F; *p*<0.01 or 0.05**), by CD4^+^ T cells, and this effect was enhanced for hMSC-treated B cells (**Figure 1C-F; *p*<0.01 or 0.05**).

**Figure 2:**
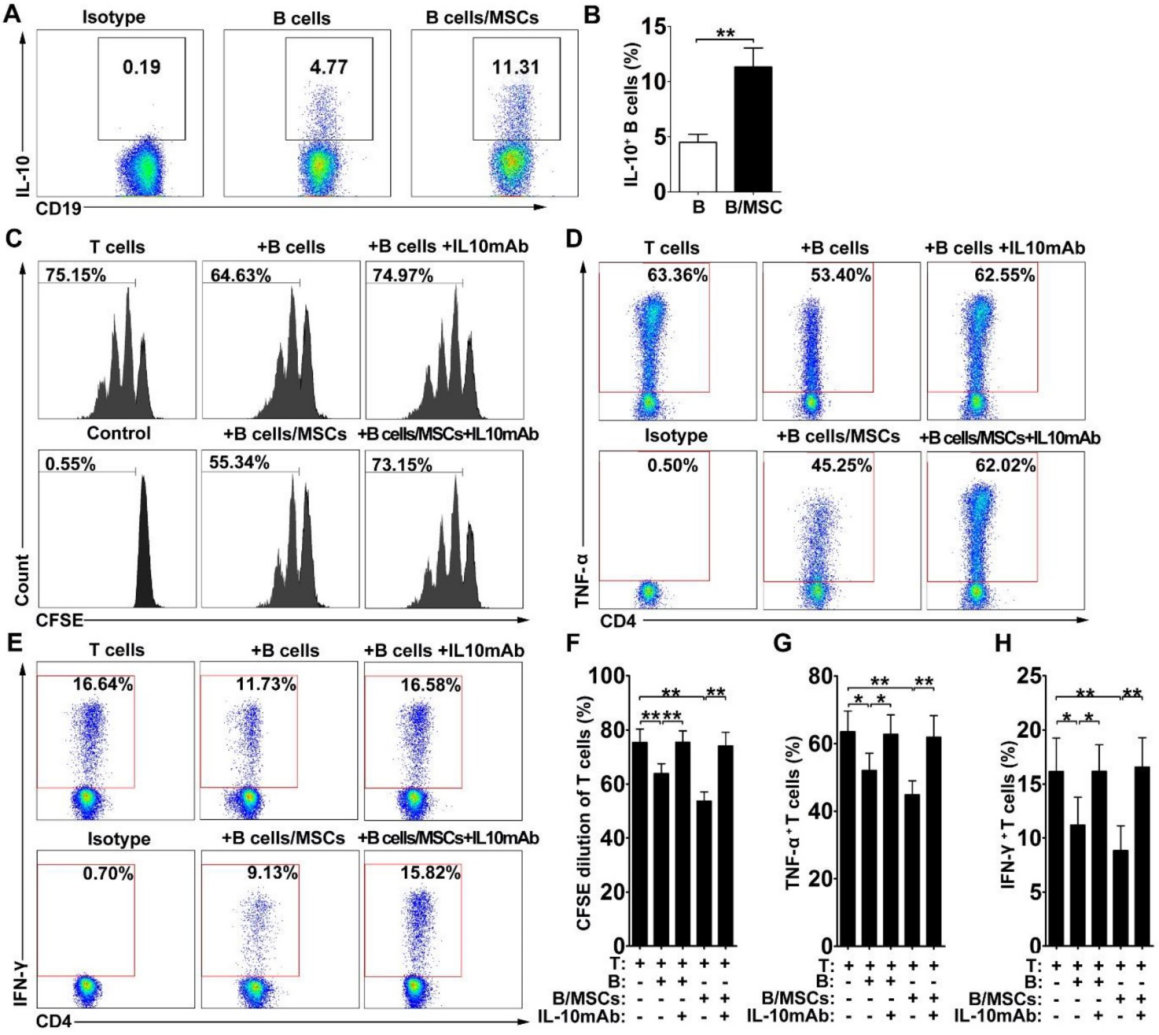
** hMSCs enhanced the immunosuppressive effect of B cells by inducing Breg cells.** B cells were cultured alone or with hMSCs in the presence of CpG ODN 2006 and CD40L for 48h, and the percentage of IL-10 producing B cells were assessed. (A) Representative plots of IL-10 production by B cells with or without hMSCs. (B) Quantification of IL-10-producing B cells. The anti-IL-10 neutralizing antibodies were added to the T/B coculture, and the alteration of T cell responses was evaluated. (C) Representative histograms of CFSE-labeled T cells' proliferation with or without IL-10 blocking. (D) Representative plots of TNF-α production by T cells with or without IL-10 blocking. (E) Representative plots of IFN-γ production by T cells with or without IL-10 blocking. (F) Quantification of CFSE-diluted T cells. (G) Quantification of TNF-α-producing T cells. (H) Quantification of IFN-γ-producing T cells. Data represent mean values ± SD of four independent experiments. *p<0.05; **p<0.01.

Since most Breg cells can express the anti-inflammatory cytokine IL-10, we assessed the production of IL-10 in our system. As shown in** Figure 2A-B,** IL-10-producing B cells were more frequent in hMSC-treated B cell cultures compared to the controls, suggesting that the presence of hMSCs may induce the production of IL-10 by B cells (***p*<0.01**).

To determine whether IL-10 is a key factor for the immunosuppressive effects of hMSC-treated Breg cells on T cells *in vitro*, we performed IL-10 neutralizing tests in our coculture system. Indeed, blockade of IL-10 apparently reversed the suppressive effects of both B cells and hMSC-treated B cells on the proliferation of CD4^+^ T cells (**Figure 2C, 2F; *p*<0.01**) and the inhibition of TNF-α (**Figure 2D, 2G; *p*<0.01 or 0.05**) and IFN-γ (**Figure 2E, 2H; *p*<0.01 or 0.05**) production in CD4^+^ T cells. These data indicate that hMSCs improve the regulatory effect of B cells by increasing IL-10 production.

### hMSCs induce CD23^+^CD43^+^ IL-10-producing B cells

**Figure 3:**
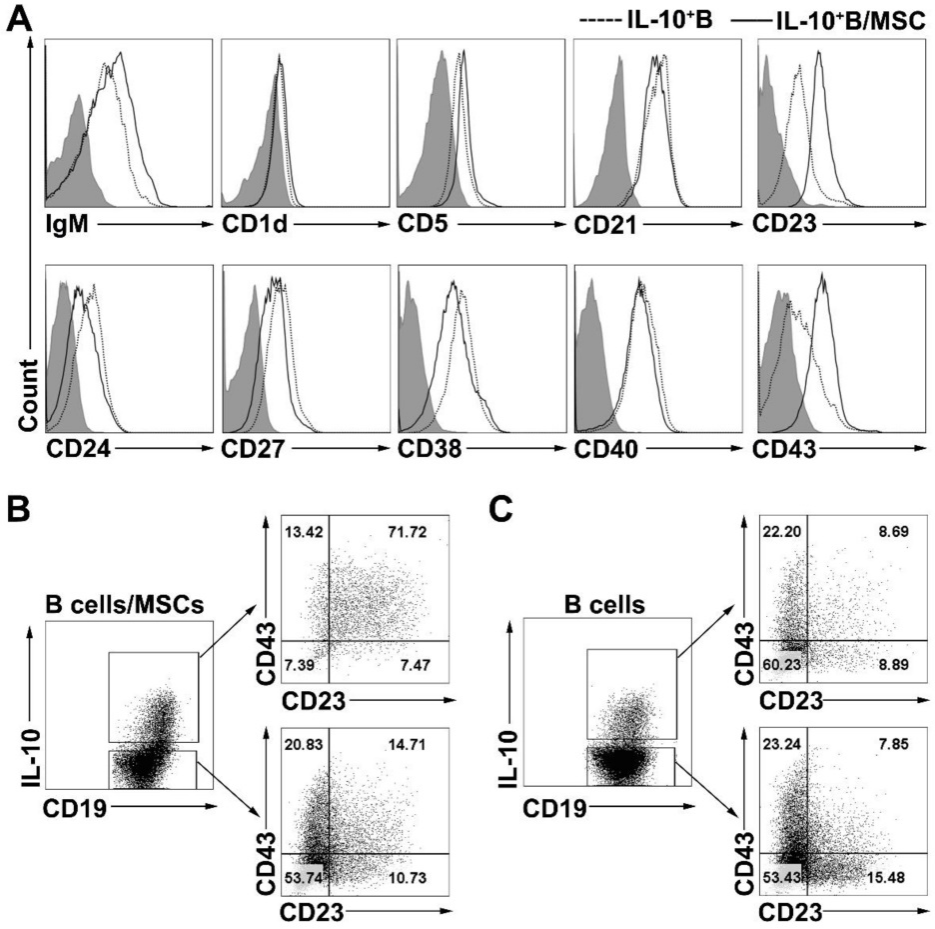
** hMSCs induced IL-10-producing B cells highly expressed CD23 and CD43.** B cells were cultured alone or with hMSCs in the presence of CpG ODN 2006 and CD40L for 48h, and the surface markers of B cells were detected. (A) Representative cell surface phenotype of B10 cells and hMSC-educated B10 cells. Filled histograms indicate isotype controls, the solid line represents the hMSC-educated IL-10^+^ B cells, and the dotted line represents the IL-10^+^ B cells without hMSCs. (B) Representative dot plots of CD23 and CD43 expression on IL10^+^ and IL10^-^ B cells cultured with hMSCs. (C) Representative dot plots of CD23 and CD43 expression on IL10^+^ and IL10^-^ B cells without hMSCs.

To phenotypically characterize the B10 cells induced by coculturing with hMSCs, we examined the cell surface phenotypes (the expressions of IgM, CD1d, CD5, CD21, CD23, CD24, CD27, CD38, CD40, and CD43) of B cells and hMSC-treated B cells. Similar to previous results 16, we found that the expression of CD5, CD23, CD24 and CD38 was notably higher on IL-10^+^ B cells than on IL-10^-^ B cells (**Figure S3A**). hMSC-treated IL-10^+^ B cells also expressed higher levels of CD5, CD23, CD24, CD38, and CD43 compared to their corresponding IL-10^-^ B cells (**Figure S3B**). To further characterize the B10 cells induced by coculturing with hMSCs, surface markers were compared between B10 cells with or without coculturing with hMSCs. As shown in **Figure 3A**, B10 cells induced by hMSCs had a substantially up- regulated CD23 and CD43 expression as compared to B10 cells without hMSCs induction. Importantly, most of the hMSC-treated IL-10-producing B cells were enriched for CD23^+^CD43^+^ B cells (**Figure 3B**) while only a small number of B10 cells without MSC treatment were CD23^+^CD43^+^ (**Figure 3C**). Taken together, hMSC-treated IL-10-producing B cells appear to predominantly represent a subpopulation of cells within the CD23^+^CD43^+^ B cell subset.

**Figure 4:**
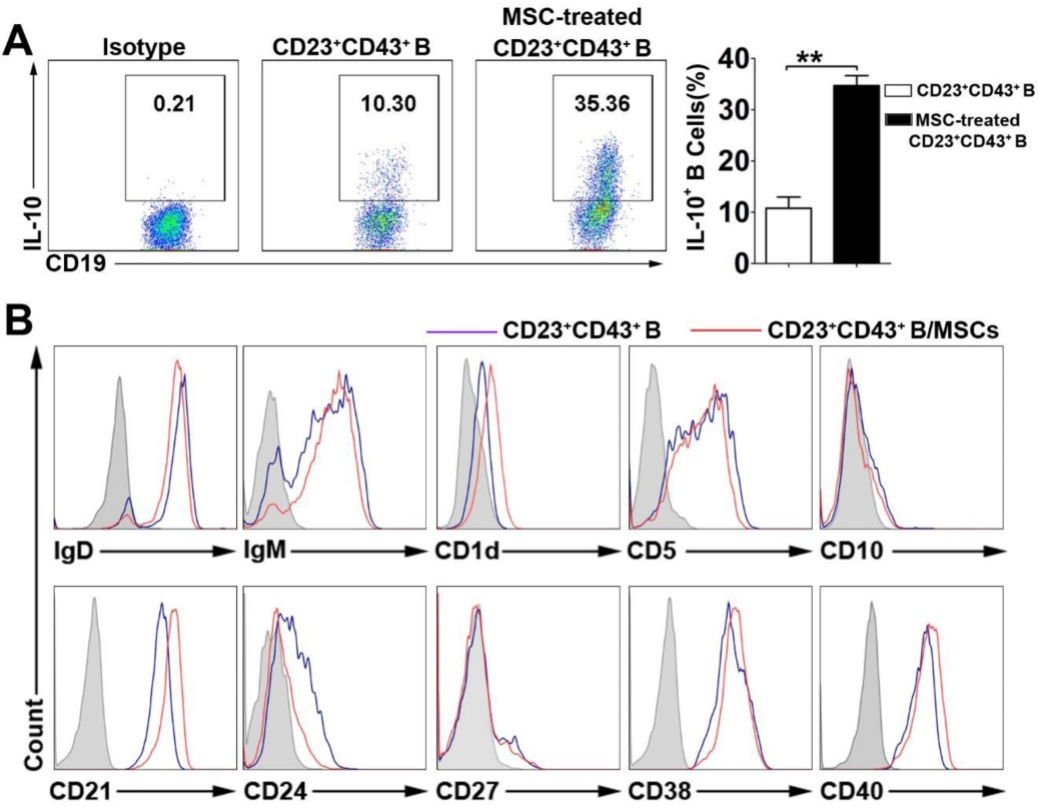
** The characteristics of hMSC-induced CD23^+^CD43^+^ B cells.** B cells were cultured with hMSCs in the presence of CpG ODN 2006 and CD40L for 48h, and the IL-10 producing ability and surface markers of hMSC-induced CD23^+^CD43^+^ B cells were detected. (A) Representative plots of IL-10 production by CD23^+^CD43^+^ B cells with or without hMSCs. (B) Representative cell surface phenotype of CD23^+^CD43^+^ B cells with or without hMSCs. Filled histograms indicate isotype controls, blue lines represent CD23^+^CD43^+^ B cells without hMSCs, and red lines represent hMSCs-educating CD23^+^CD43^+^ B cells. Data represent mean values ± SD of five independent experiments. *p<0.05; **p<0.01.

To define the characteristics of the hMSC-treated Breg cell subset, we gated on the CD23^+^CD43^+^ B cells and analyzed their capacity of IL-10 production with or without hMSC treatment. As expected, hMSC- treated CD23^+^CD43^+^ B cell subset showed the regulatory B cell characteristics as robust IL-10 secretion, which was significantly higher than that in CD23^+^ CD43^+^ B cells without coculturing with hMSCs (**Figure 4A; *p*<0.01**). Furthermore, this B cell subset highly expressed IgD, IgM, CD5, CD21, CD38 and CD40, but without significant difference from the CD23^+^CD43^+^ B cells without hMSCs coculture** (Figure 4B)**.

### CD23^+^CD43^+^ B cells regulate the T cell response via IL-10

**Figure 5:**
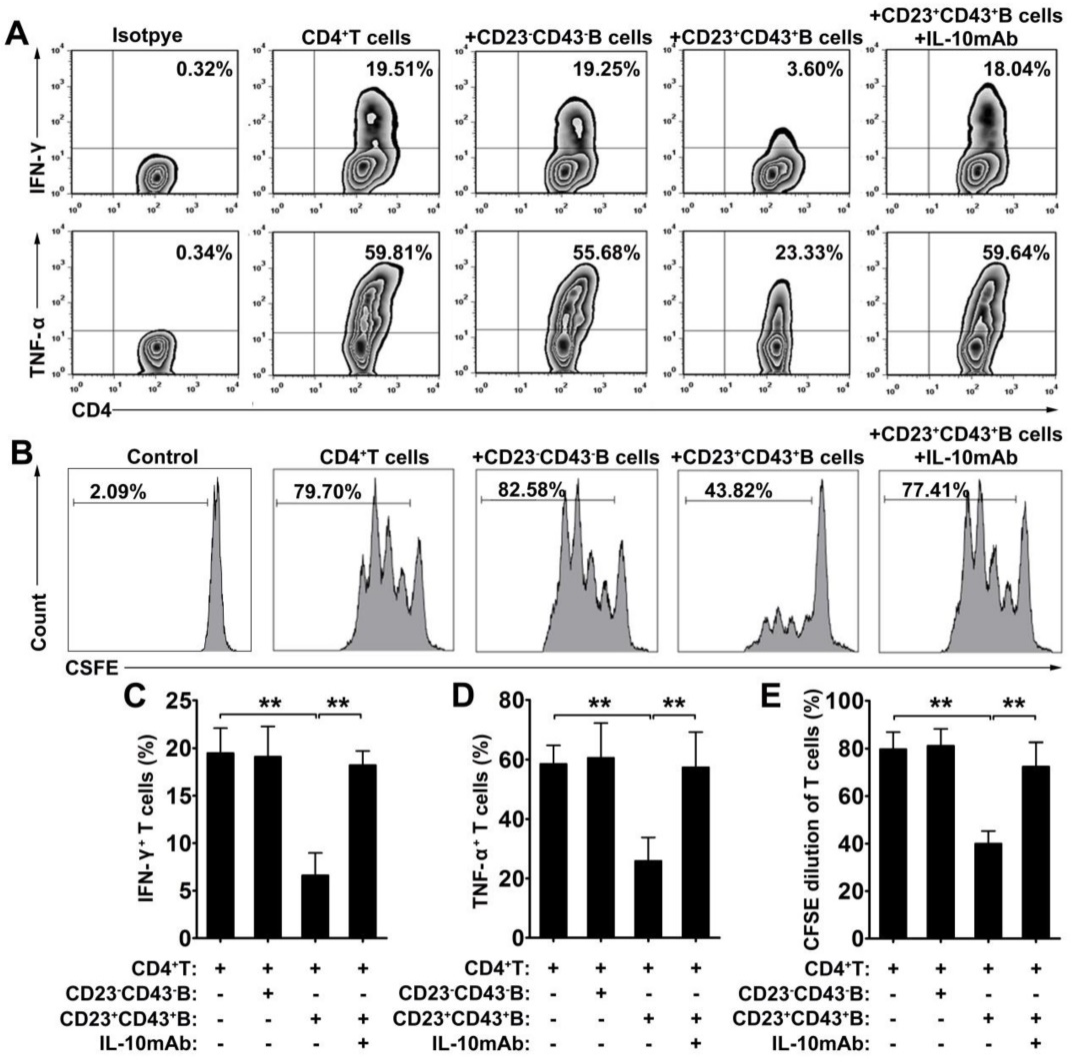
** CD23^+^CD43^+^ B cells inhibited the T cell response dependent on IL-10 production.** B cells were cultured with hMSCs in the presence of CpG ODN 2006 and CD40L for 48h, then CD23^+^CD43^+^ B cells and CD23^-^CD43^-^ B cells were isolated from hMSC-educated B cells and cultured with T cells. (A) Representative plots of cytokine secretion. CD23^+^CD43^+^ B cells, but not CD23^-^CD43^-^ B cells, suppressed the TNF-α and IFN-γ production by T cells that was reversed by anti-IL-10mAb. (B) Representative histograms of T cell proliferation. CD23^+^CD43^+^ B cells, but not CD23^-^CD43^-^ B cells, suppressed the proliferation of CFSE-labeled T cells that was reversed by anti-IL-10mAb. (C) Quantification of IFN-γ-producing T cells. (D) Quantification of TNF-α-producing T cells. (E) Quantification of CFSE-diluted T cells. Data represent mean values ± SD of four independent experiments. *p<0.05; **p<0.01.

To further investigate the immunoregulatory function of hMSC-treated CD23^+^CD43^+^ B cells, we cocultured B cells with hMSCs for 2 days, then purified CD23^+^CD43^+^ B and CD23^-^CD43^-^ B cells **(Figure S4)** and cultured the corresponding cells with CD4^+^ T cells. The results revealed that coculturing with CD23^+^CD43^+^ B cells inhibited the secretion of IFN-γ and TNF-α by CD4^+^ T cells, whereas CD23^-^ CD43^-^ B cells did not affect the expression of these cytokines by CD4^+^ T cells (**Figure 5A, C-D; *p*<0.01**). Moreover, CD23^+^CD43^+^ B cells, but not CD23^-^CD43^-^ B cells, significantly suppressed CD4^+^ T cell proliferation (**Figure 5B, E; *p*<0.01**). The addition of anti-IL-10mAb to cultures containing CD23^+^CD43^+^ B cells largely rescued the IFN-γ and TNF-α production (**Figure 5A, C-D; *p*<0.01**) and proliferation (**Figure 5B, E; *p*<0.01**) of CD4^+^ T cells. These data suggest that hMSC-treated CD23^+^CD43^+^ Breg cells can inhibit the inflammatory cytokine secretion and proliferation of T cells through an IL-10-dependent pathway.

### hMSCs increase CD23^+^CD43^+^ Breg cells by promoting their proliferation and conversion from CD23^-^CD43^-^ B cells via cell-to-cell contact and COX-2/PGE2 pathway

**Figure 6:**
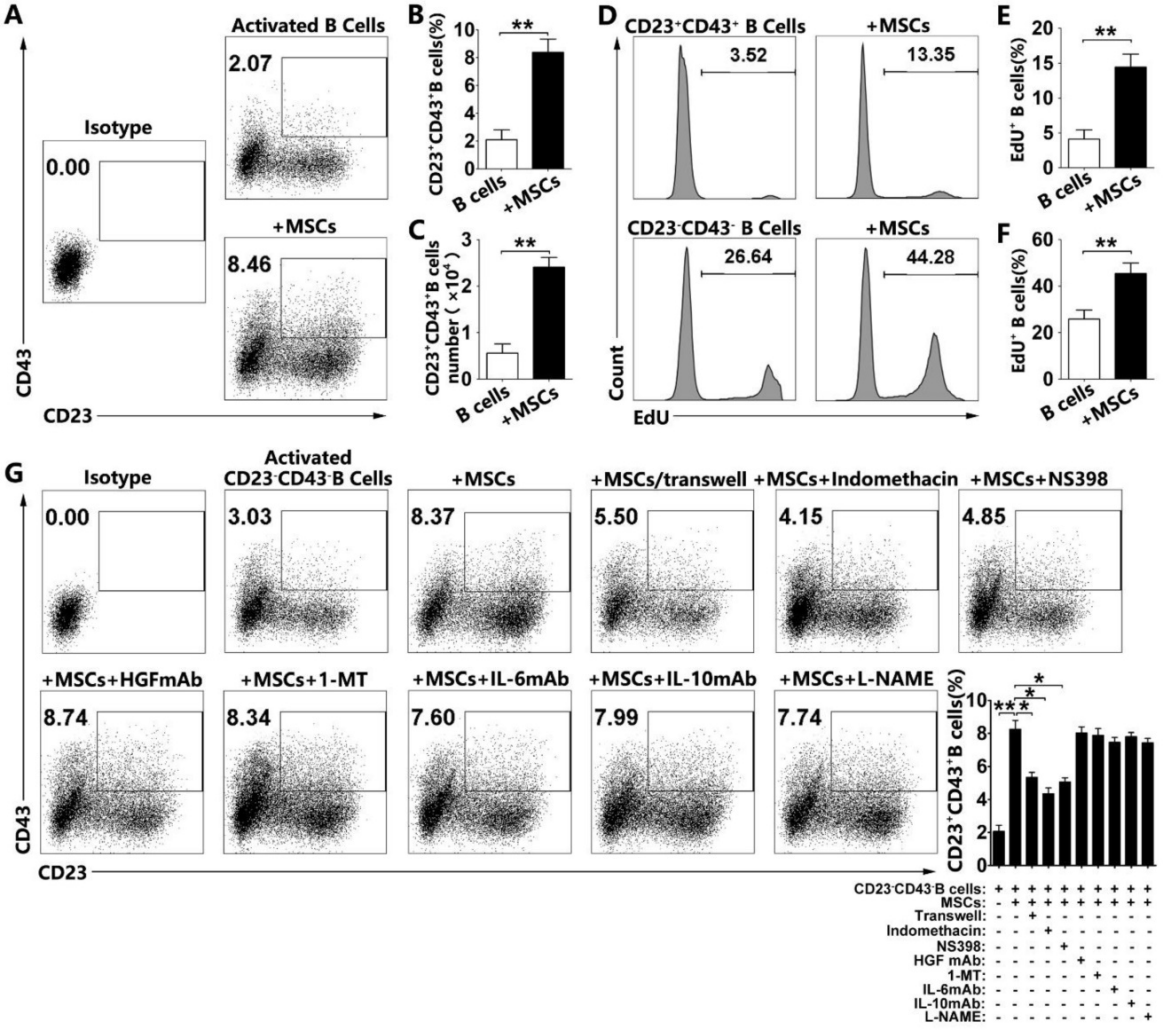
** hMSCs induced CD23^+^CD43^+^ B cells by promoting their proliferation and conversion from CD23^-^CD43^-^ B cells by cell-to-cell contact and COX2/PGE2 pathway.** Purified CD19^+^B cells or CD19^+^CD23^-^CD43^-^ B cells were cultured alone or with hMSCs in the presence of CpG ODN 2006 and CD40L for 48h. (A) Representative plots of alteration in percentages of CD23^+^CD43^+^B cells in B cells with or without hMSCs. (B) Quantification of CD23^+^CD43^+^ B cell numbers. (C) Quantification of CD23^+^CD43^+^ B cell numbers. (D) Representative histogram of the proliferation of CD23^+^CD43^+^ and CD23^-^CD43^-^ B cells alone or with hMSCs. (E) Quantification of the proliferation of CD23^+^CD43^+^ B cells. (F) Quantification of the proliferation of CD23^-^CD43^-^ B cells. (G) Representative plots of increase in percentages of CD23^+^CD43^+^ B cells generated from CD23^-^CD43^-^ B cells, quantification of CD23^+^CD43^+^ B cells. Data represent mean values ± SD of five independent experiments. *p<0.05; **p<0.01.

To examine how hMSCs induce regulatory CD23^+^CD43^+^ B cells, we first tested the proportions of CD23^+^CD43^+^ B cells in the culture. Our results confirmed that hMSCs significantly increased the number of CD23^+^CD43^+^ B cells (**Figure 6A, 6B; *p*<0.01**). Similarly, the number of the CD23^+^CD43^+^ B cells was dramatically increased after coculturing with hMSCs **(Figure 6C; *p*<0.01)**. Also, we found that hMSCs could promote the proliferation of both CD23^+^CD43^+^ B cells and CD23^-^CD43^-^ B cells. Similarly, the EdU^+^CD23^+^CD43^+^ B cells with hMSCs were four times higher than those without hMSCs, but EdU^+^CD23^-^CD43^-^ B cells with hMSCs were fewer than two times as those without hMSCs (**Figure 6D-F; p<0.01**).

To address the possibility of conversion from CD23^-^CD43^-^ B cells to CD23^+^CD43^+^ B cells, we sorted the CD23^-^CD43^-^ B cells for further studies with over 98% efficiency (**Figure S5**). As expected, a fraction of CD23^+^CD43^+^ B cells was generated from CD23^-^CD43^-^ B cells after activation (**Figure 6G**). Importantly, the numbers of CD23^+^CD43^+^ B cells were significantly higher in hMSC-treated CD23^-^CD43^-^ B cells than those without hMSCs (**Figure 6G; *p*<0.01**). These findings suggested that CD23^+^CD43^+^ Breg cells could be generated from CD23^-^CD43^-^ B cells, and hMSCs dramatically increased CD23^+^CD43^+^ Breg cells likely via promoting their conversion from CD23^-^CD43^-^ B cells. Taken together, hMSCs increase CD23^+^CD43^+^ Breg cells both by promoting their proliferation and conversion from CD23^-^CD43^-^ B cells.

To gain further insight into the mechanism of hMSC-mediated increase of CD23^+^CD43^+^ B cells, we first used the Transwell assay to investigate whether hMSCs increased these cells via cell-to-cell contact. Although hMSCs in the Transwell culture could increase the percentage of CD23^+^CD43^+^ B cells, it was still much less than the percentage of those in direct contact with hMSCs (**Fig. 6b; *p*<0.05**). We speculated that both cell-to-cell contact and paracrine pathway are involved in the regulation of CD23^+^CD43^+^ B cells by hMSCs. We then tested several potential mediators known to be involved in MSC-mediated immunomodulation 11, 33 and the induction of IL-10- producing regulatory T cells 34, 35. These mediators included indomethacin (a non-specific inhibitor for COX-2/PGE2), NS398 (a specific inhibitor for COX-2/ PGE2), 1-MT (a specific inhibitor for IDO), anti-HGF antibody, anti-IL-6 antibody, anti-IL-10 antibody, and L-NAME (a nonselective inhibitor of NOS). Among these targets, only indomethacin and NS398 partially reversed the hMSC-induced increase in the frequency of CD23^+^CD43^+^ B cells (**Figure 6G; *p*<0.05**). It appears that both the cell-to-cell contact and COX-2/PGE2 pathway participate in the process of hMSCs regulating the increase of CD23^+^CD43^+^ B cells.

To explore the possible signaling pathways that might mediate the conversion from CD23^-^CD43^-^ B cells to CD23^+^CD43^+^ B cells during coculture with hMSCs, we purified CD23^-^CD43^-^ B cells, and cultured them with hMSCs in the presence of CpG ODN 2006 and CD40L. After 36h, hMSC-induced CD23^+^CD43^+^ B cells were purified, and the transcriptome analysis was performed. The purified CD23^-^CD43^-^ B cells before co-culturing with hMSCs served as the control. The transcriptome analysis identified upregulation of 3000 genes and downregulation of 2000 genes in these cells in hMSC-treated CD23^+^CD43^+^ B cell subpopulation. GO analysis indicated that the up-regulated genes were highly enriched in the cell cycle, signaling by receptor tyrosine kinases (RTKs), Wnt signaling pathway, and PI3K-AKT signaling pathway (**Figure S6**).

### hMSC-treated CD23^+^CD43^+^ Breg cells effectively inhibit colon inflammation in TNBS-induced colitis

**Figure 7:**
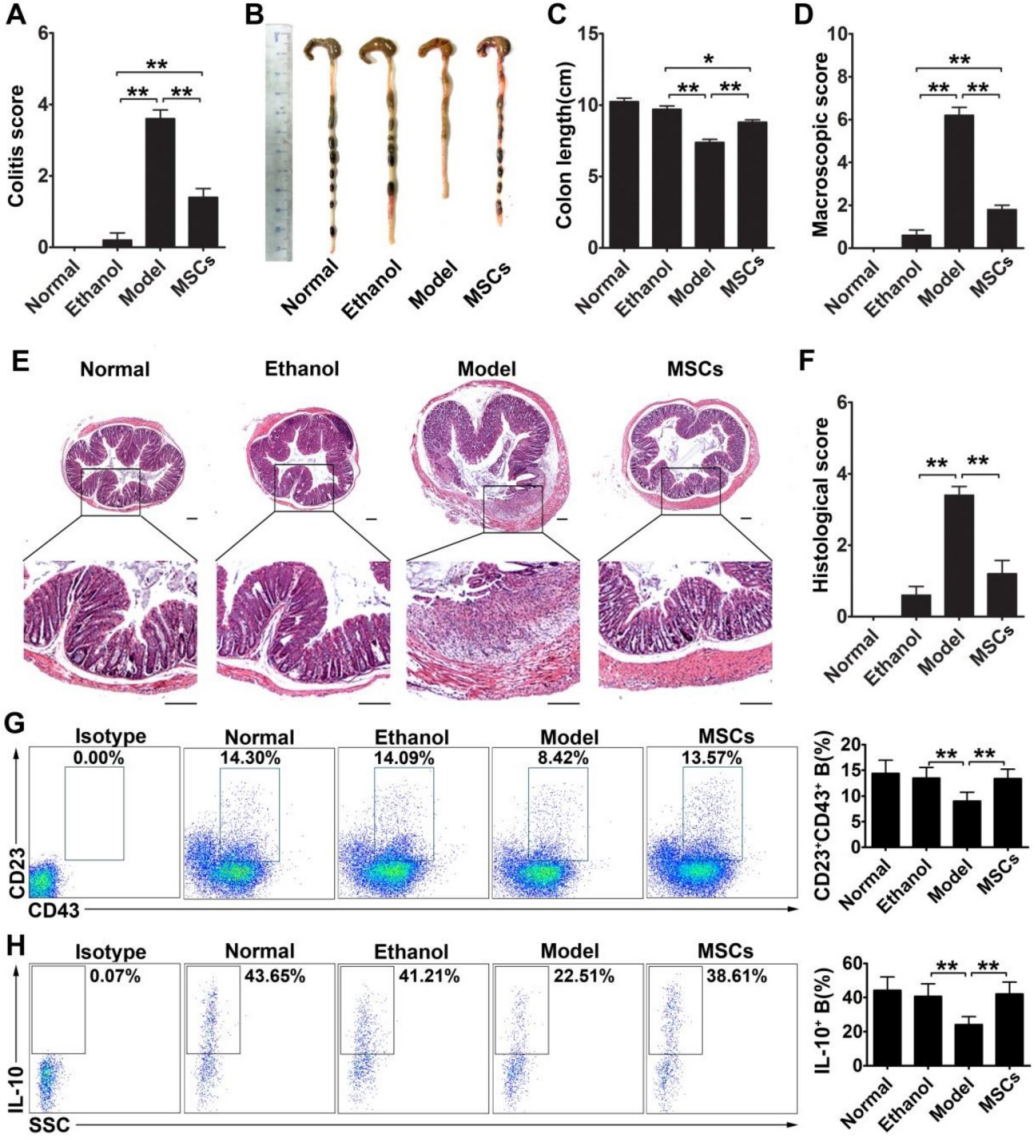
** hMSCs alleviated colon inflammation in TNBS-induced colitis and CD23^+^CD43^+^ B cells recovery in the peritoneal cavity.** Colitis was induced by TNBS in mice, which received the intraperitoneal injection of hMSCs or saline. Mice were euthanized on day 3 after treatment. (A) Colitis score of mice. (B) Representative colonic length of mice. (C) Quantification of the colonic length of mice. (D) Macroscopic damage score of colons. (E) Representative histological changes of colons. Scale bar=200 μm. (F) Histological score of colons. (G) Alteration in percentages of CD23^+^CD43^+^ B cells in the peritoneal cavity. (H) Changes in IL-10 production of CD23^+^CD43^+^ B cells in the peritoneal cavity. Data represent mean values ± SD of five mice per group. *p<0.05; **p<0.01.

To determine the effect of CD23^+^CD43^+^ Breg cells *in vivo*, we explored whether these cells could contribute to the therapeutic effect of hMSCs on mice with TNBS-induced colitis. As shown in **Figure 7A-F**, intraperitoneal injection of hMSCs significantly alleviated the severity of colonic inflammation compared with the control group (**Figure 7B-C; *p*<0.01**). Upon histological examination, we observed the destruction of the entire epithelium, severe submucosal edema, and scattered infiltration of inflammatory cells in the submucosa in the colons of TNBS-treated mice (**Figure 7E**). In the hMSCs-treated group, however, the mucosal destruction and edema of the submucosa were ameliorated compared with the controls (**Figure 7E**). Furthermore, the serum TNF-α, IFN-γ, IL-1β, IL-6, and IL17 were significantly increased in the colitic mice, and hMSCs treatment could reduce these inflammatory cytokines, suggesting that hMSCs alleviated the inflammatory responses in colitis mice (**Figure S7**). To further confirm that these improvements in colitis mice were induced by hMSCs, we used human dermal fibroblasts (hDFs), commonly used as controls for human MSCs, to exclude the interference of xenogeneic response to hMSCs. As expected, hMSCs, rather than hDFs, significantly alleviated the colitis (**Figure S8**). These observations further supported that hMSCs themselves alleviated TNBS-induced colitis in mice.

**Figure 8:**
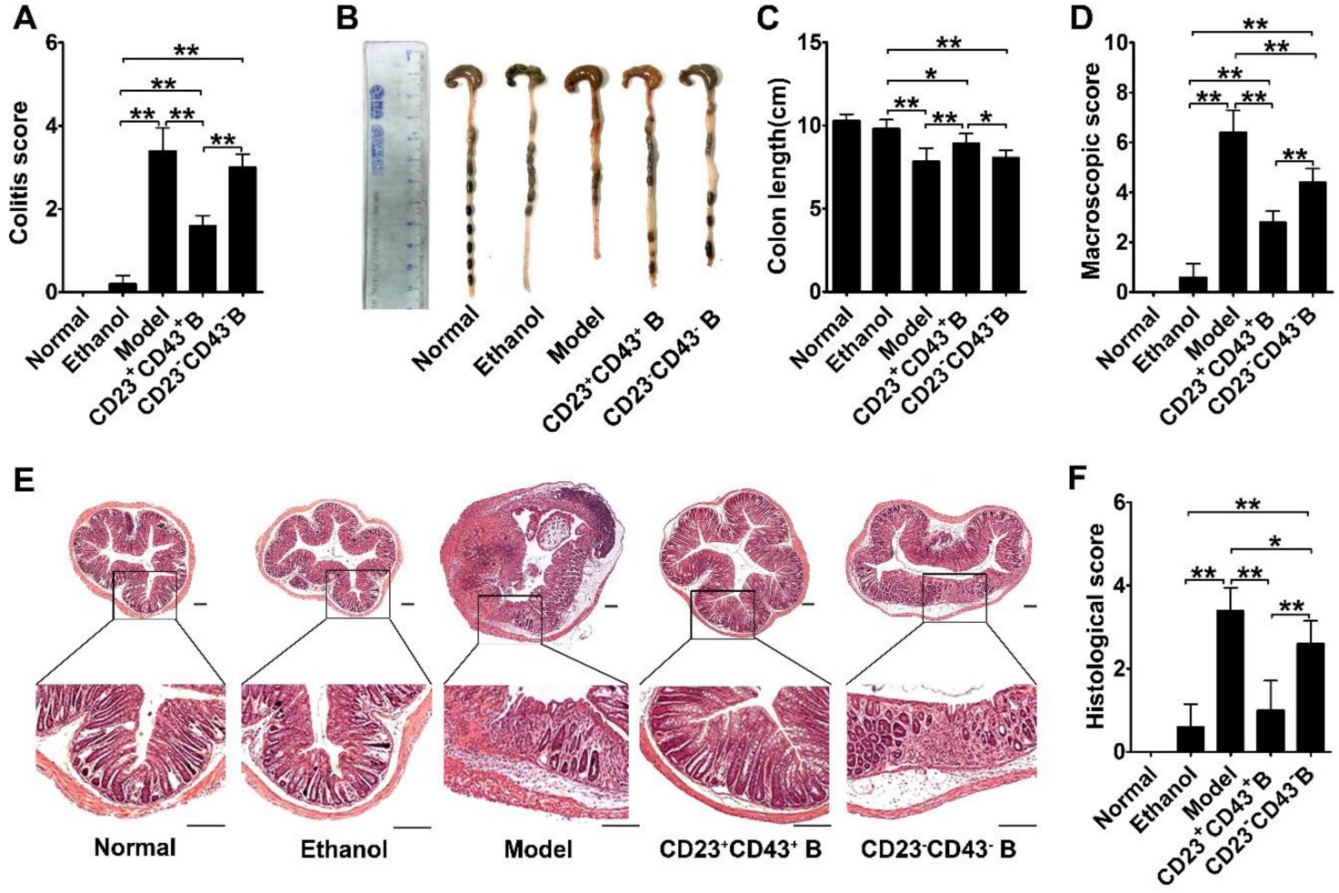
** CD23^+^CD43^+^ B cells adoptive transfer greatly reduced the severity of colitis.** Colitis was induced by TNBS in mice by intraperitoneal injection of purified CD23^+^CD43^+^ B cells or CD23^-^CD43^-^ B cells. Mice were euthanized on day 3 after treatment. (A) Colitis score of mice. (B) Representative colonic length of mice. (C) Quantification of the colonic length of mice. (D) Macroscopic damage score of colons. (E) Representative histological changes of colons. Scale bar=200 μm. (F) Histological score of colons. Data represent mean values ± SD of five mice per group. *p<0.05; **p<0.01.

Furthermore, we found that the significant improvements by hMSC treatment were associated with increasing the CD23^+^CD43^+^ Breg cell population and raising IL-10 levels (**Figure 7G-H; *p*<0.01**). We isolated CD23^+^CD43^+^ and CD23^-^CD43^-^ B cells from the peritoneal cells of mice and examined their contribution to the therapeutic effects of hMSCs on colitis. The adoptive transfer of CD23^+^CD43^+^ B cells to colitic mice significantly reduced the severity of colitis (**Figure 8A; *p*<0.01**), restored colon length (**Figure. 8B-C; *p*<0.01**), and lessened the histological damage to the colon (**Figure. 8D, F**). However, adoptive transfer of CD23^-^CD43^-^ B cells failed to improve the symptoms (**Figure 8A-F; *p*<0.01 or *p*<0.05**).

**Figure 9:**
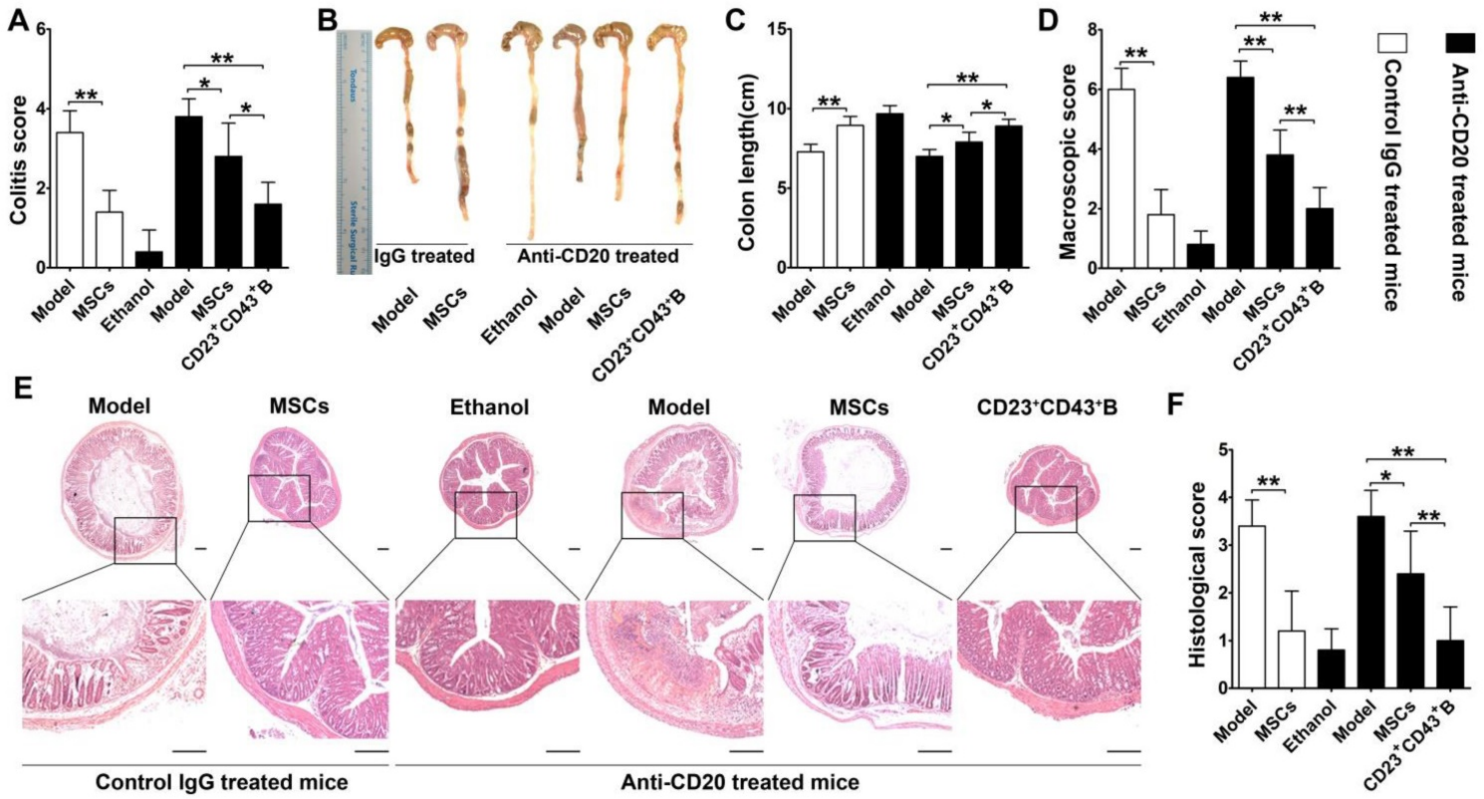
** CD23^+^CD43^+^ B cells but not hMSCs significantly mitigated colitis in B cell-depleted mice.** Colitis was induced by TNBS in B cell-depleted mice, which received the intraperitoneal injection of hMSCs, CD23^+^CD43^+^ B cells, or saline. Mice were euthanized on day 3 after treatment. (A) Colitis score of mice. (B) Representative colonic length of mice. (C) Quantification of the colonic length of mice. (D) Macroscopic damage score of colons. (E) Representative histological changes of colons. Scale bar=200 μm. (F) Histological score of colons. Data represent mean values ± SD of five mice per group. *p<0.05; **p<0.01.

To further confirm the role of hMSC-treated CD23^+^CD43^+^ B cells in alleviating colitis, we investigated these therapeutic effects in B cell-depleted mice. For this, mice were i.v. injected with anti-mouse CD20 antibody which barely detected B cells in peripheral blood and peritoneal cavity at 1 week after treatment **(Figure S9)**. These B cell-depleted mice were then treated with TNBS to induce colitis, intraperitoneally injected with hMSCs, CD23^+^CD43^+^ B cells, and saline. As shown in **Figure 9**, hMSCs could significantly alleviate the colitis in B cell-sufficient mice (treated with the Rat IgG2b, κ, an isotype for anti-mouse CD20 as control). However, hMSCs minimally improved the clinical parameters of colitis in mice when B cells were depleted by anti-mouse CD20 antibody. Adaptive transfer of CD23^+^CD43^+^ B cells significantly reduced the severity of colitis **(Figure 9; *p*<0.01 or *p*<0.05)**, indicating that CD23^+^CD43^+^ B cells might be the hMSC-induced Breg cells that can effectively mitigate the mucosal inflammatory response.

## Discussion

Previous studies have demonstrated that MSCs have immunoregulatory functions and can be used to treat a wide range of immune-mediated diseases 4. Although MSCs are known to modulate the immune response by interacting with cells of both innate and adaptive immunity, the underlying mechanisms and cellular components involved are not yet known. Mounting evidence suggests that MSCs might not act directly as immuno-regulators, but rather they might re-educate immune cells to generate regulatory immune cells with tolerogenic properties 33. These regulatory immune cells, including Tregs, Bregs, regulatory antigen presenting cells (APCs), and natural killer cells (NK) cells, together create a tolerogenic environment suitable for modulating the immune response 36.

We previously showed that hMSCs alleviated experimental colitis, altered the imbalances of Treg/Th1/Th17 cells, and strikingly increased the number of IL-10-producing CD5^+^ Breg cells 20. Moreover, our clinical study demonstrated that CD5^+^ Breg cells play an important role in regulating the immune response via IL-10 19. In the present study, we confirmed that activated B cells could alter the T cell response by regulating proinflammatory cytokine production and suppressing proliferation, and that this effect was largely dependent on IL-10. We also showed that coculturing with hMSCs further enhanced these regulatory activities of B cells by increasing the number of IL-10-producing B cells. It is well known that MSCs recruit and induce regulatory T cells 37, and that these cells play a critical role in MSC-mediated immuno-modulation. Similarly, our present results support the idea that MSCs may also induce regulatory B cells that confer their regulatory effects via IL-10. Our findings offer new insights into the effects of MSCs on B cells and the therapeutic mechanism through which MSCs impart their beneficial effects in clinical studies.

Breg cells are known to be heterogeneous, and several distinct regulatory B cell subsets have been identified. Here, we found that the hMSCs induced IL-10-producing CD23^+^CD43^+^ B cells with an unconventional phenotype. They shared some phenotypic features with multiple previously described Breg cell subsets 21-28 in that they were positive for IgM, CD5, CD24 and CD38. However, they differed in their expression of CD23 and CD43. CD23 is a low-affinity IgE receptor that is known to regulate the synthesis of IgE by binding IgE/antigen complexes 38 and is reportedly expressed on activated B cells 39, 40. In mice, CD23 has been used as a surface marker for two subsets of Breg cells: transitional 2 marginal zone precursor (T2-MZP) Breg cells and GM-CSF-IL-15- induced Breg cells 41. CD43, which is also known as leukosialin, is expressed on murine and human pluripotent hematopoietic stem cells 42 and on almost all hematopoietic cell lineages 43, 44. CD43 is not expressed on resting B cells; instead, it is induced during the activation and differentiation of human B cells 45, 46. Moreover, a recent study demonstrated that CD43 is expressed on a specific type of B1 cell population (CD20^+^CD27^+^CD43^+^CD70^-^) in human umbilical cord and adult peripheral blood 47, 48. Considering that Breg cells are a subset of antigen- experienced B cells 49, it is conceivable that they possess the activation-related markers, CD23 and CD43, which are reported markers for B10 cells 50. Our results also supported this notion; we found that CD23^+^CD43^+^ B cells could be generated from the CD23^-^CD43^-^ B cells after activation, and hMSCs treatment likely promoted their proliferation and conversion from CD23^-^CD43^-^ B cells. Indeed, we observed that the MSC-treated IL-10-producing B cells were present in the CD23^+^CD43^+^ B cell subset, suggesting that the CD23^+^CD43^+^ phenotype may define a subpopulation of MSC-treated IL-10- producing B cells. Although CD23^+^CD43^+^ B cells shared several other surface markers that may be used to define Breg cells, they are likely be the most representative subset of MSC-treated IL-10-producing B cells.

Growing evidence suggests that IL-10 producing Breg cells act as potent regulators in many diseases, including inflammatory bowel disease (IBD) 14. IL-10 producing Breg cells were decreased in mice or humans with colitis 51, 52. It has been reported that human MSCs can regulate the immune responses in immunocompetent mice by a variety of mechanisms that are similar to those employed by mouse MSCs 53. Therefore, to investigate whether human MSCs improved inflammation-related diseases via increasing IL-10 producing Bregs, we employed the murine colitis model, which has been widely used to evaluate the immunomodulatory properties of human MSCs 54-56. Our data suggested that CD23^+^CD43^+^ Breg cells were associated with the efficacy of administered hMSCs against IBD. Administration of hMSCs via peritoneal injection increased the number of CD23^+^CD43^+^ Breg cells which produced an elevated level of IL-10. More importantly, when CD23^+^CD43^+^ B cells were isolated and adoptively transferred into TNBS-induced IBD mice, these Breg cells clearly reduced disease symptoms. Furthermore, hMSCs showed low efficiency in improving the colitis in B cell-depleted mice while adaptive transfer of CD23^+^CD43^+^ B cells significantly ameliorated the clinical and histopathological severity of induced colonic inflammation and restored the injured gastrointestinal mucosal tissues. These results further demonstrated that hMSCs mitigated the experimental colitis partially through B cells, especially the CD23^+^CD43^+^ B cells.

Taken together, our present findings provide new data defining the phenotype and suppressive activity of hMSC-treated CD23^+^CD43^+^ Breg cells which may be an important regulatory B cell subset responsible for the ability of hMSCs to control inflammation-related diseases.

## Abbreviations

MSCs: mesenchymal stem cells
Breg: regulatory B cells
FACS: fluorescence-activated cell sorting
IBD: inflammatory bowel disease
Tregs: regulatory T cells
T2-MZP: T2 marginal zone precursor
ICCS: intracellular cytokine staining
BFA: brefeldin A
PMA: phorbol 12-myristate 13-acetate
IDO: indoleamine 2,3-dioxygenase
COX-2: cyclooxygenase-2
PGE2: prostaglandin E2
1-MT: 1-methyl-tryptophan
NOS: nitric oxide synthase
TNBS: trinitrobenzenesulfonic acid
