# Supplementary material for: Human Mesenchymal Stem Cell-Treated Regulatory CD23+CD43+ B Cells Alleviate Intestinal Inflammation
Source: Theranostics. 2019 Jun 24;9(16):4633–47. doi: 10.7150/thno.32260 (PMC6643430; doi:10.7150/thno.32260)
Supplement: Supplementary file 1 — Supplementary figures and tables. [file thnov09p4633s1.pdf]

Human mesenchymal stem cell-treated regulatory $CD23^+CD43^+$ B cells alleviate intestinal inflammation

Xiaoyong Chen, Chuang Cai, Dijing Xu, Qiuli Liu, Shuwei Zheng, Longshan Liu, Gang Li, Xiaoran Zhang, Xiaoping Li, Yuanchen Ma, Li Huang, Jieying Chen, Jiahao Shi, Xin Du, Wenjie Xia, Andy Peng Xiang, Yanwen Peng

Supplementary Figures

Figure S1. Characterization of hMSCs.

Figure S2. Dynamic observation of T cells proliferation.

Figure S3. hMSCs inducing IL-10-producing B cells highly express CD23 and CD43.

Figure S4. The sort gating for the $CD23^+CD43^+$ and $CD23^-CD43^-$ B cells.

Figure S5. Assessment of the purity of isolated $CD23^-CD43^-$ B cells.

Figure S6. GO analysis of the DEGs of MSC-induced $CD23^+CD43^+$ B cells

Figure S7. Alteration of serum inflammatory cytokines in colitis mice treated with hMSCs.

Figure S8. Fibroblast could not alleviate the colitis mice.

Figure S9. Alteration of B cells before and after anti-CD20 antibody administration.

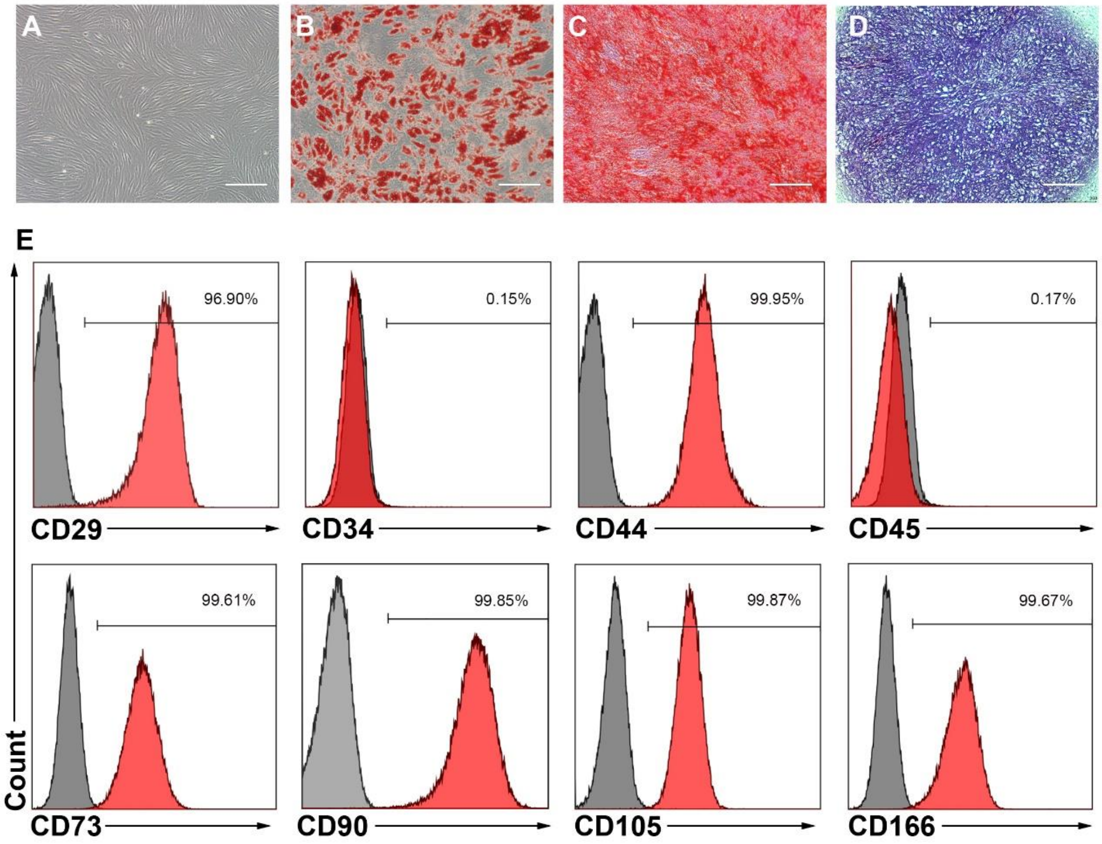

Figure S1. Characterization of hMSCs

Morphology of human MSCs (passage 5) was revealed by phase contrast microscopy (A). Oil O staining showed adipogenic differentiation of human MSCs (B). Alizarin red S staining showed osteogenic differentiation of human MSCs (C). Toluidine blue staining showed chondrogenic differentiation of human MSCs (D). Flow cytometric analysis of cell surface markers of human MSCs (E). Scale bar=200 μm. Filled grey histograms indicate isotype controls, red histograms represent the hMSCs.

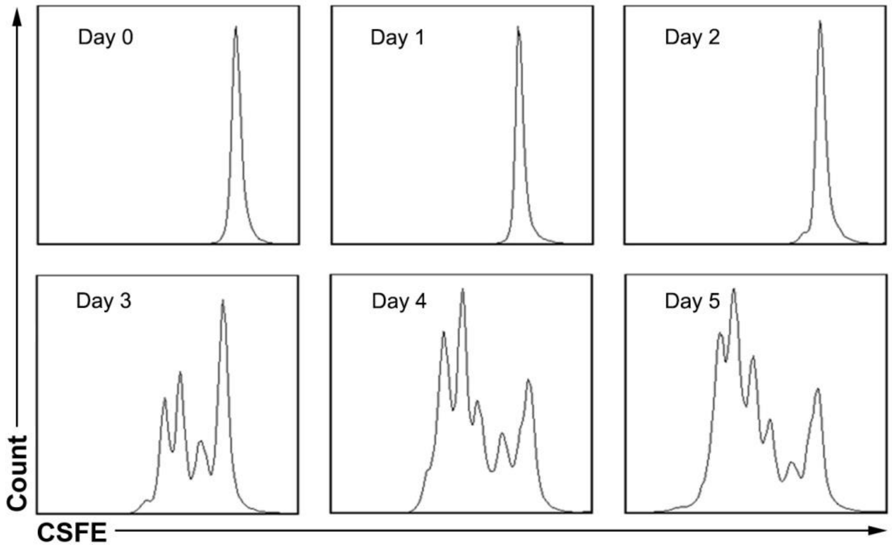

Figure S2. Dynamic observation of T cell proliferation

Purified $CD4^+$ T cells were labeled with CFSE and cultured in the presence of anti-CD3mAb and anti-CD28mAb. T cell proliferation was evaluated by flow cytometry. Representative histograms show dynamic changes of CFSE dilution every day.

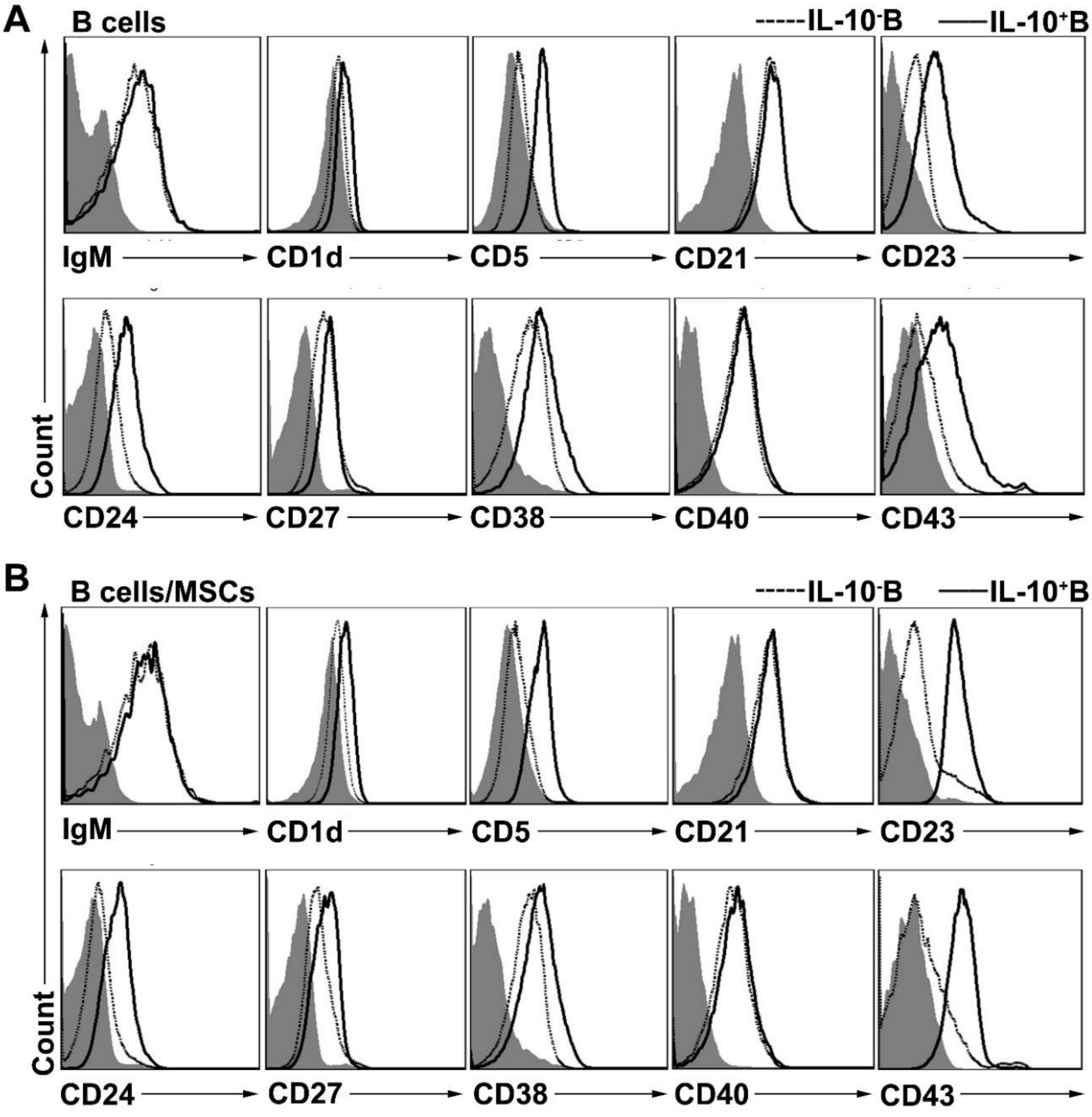

Figure S3. hMSC induced IL-10-producing B cells highly expressed CD23 and CD43

B cells were cultured alone or with hMSCs in the presence of CpG ODN 2006 and CD40L for 48h, and the surface marker of B cells were detected. (A) Representative cell surface phenotype of B cells cultured alone. (B) Representative cell surface phenotype of hMSCs-educated B cells. Filled histograms indicate isotype controls, the solid line represents the IL-10^{+} B cells, and the dotted line represents the IL-10^{-} B cells.

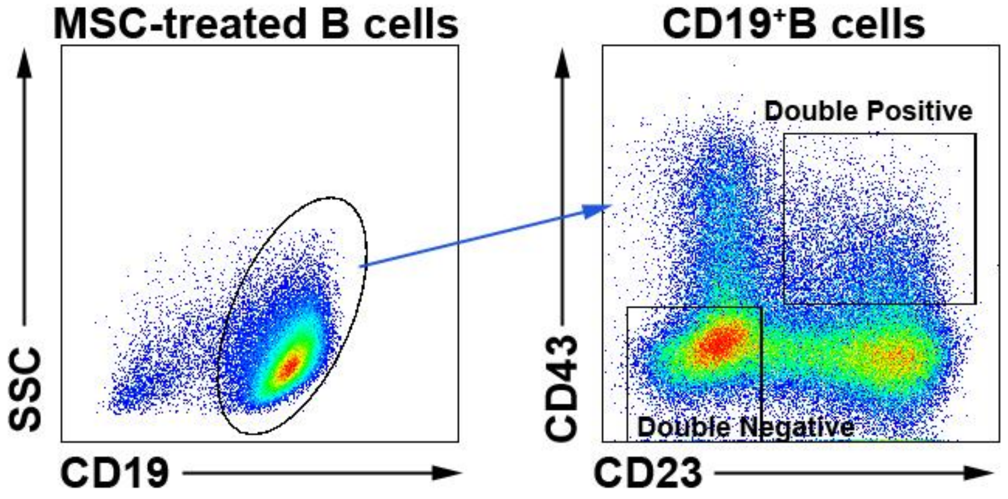

Figure S4. Sort gating for CD23^{+}CD43^{+} and CD23^{-}CD43^{-} B cells

Purified CD19^{+}B cells were cocultured with hMSCs in the presence of CpG and CD40L for 2 days. Then, B cells were harvested, and stained with antibodies including anti-CD19, anti-CD23, and anti-CD43. While sorting by flow cytometry, lymphocytes were gated, then CD19^{+} B cells among lymphocytes were gated followed by CD23^{+}CD43^{+} B and CD23^{-}CD43^{-} B cells within CD19^{+} B cells. Representative dot plots show the sort gating for the CD23^{+}CD43^{+} and CD23^{-}CD43^{-} B cells.

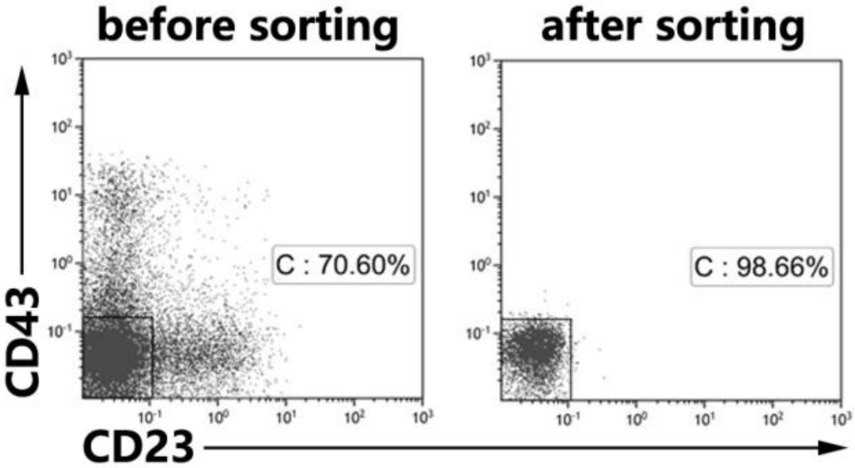

Figure S5. Assessment of the purity of isolated CD23⁻CD43⁻ B cells

PBMCs from healthy donors were stained with antibodies including anti-CD19, anti-CD23, and anti-CD43. While sorting by flow cytometry, lymphocytes were gated, then $CD19^+$ B cells among lymphocytes were gated followed by the $CD23^-CD43^-$ B cells within $CD19^+$ B cells. The representative plot of $CD23^-CD43^-$ B cells before sorting is shown in the left panel. After sorting, the sorted $CD23^-CD43^-$ B cells were subjected to purity testing. The representative plot of purified $CD23^-$ $CD43^-$ B cells is shown in the right panel.

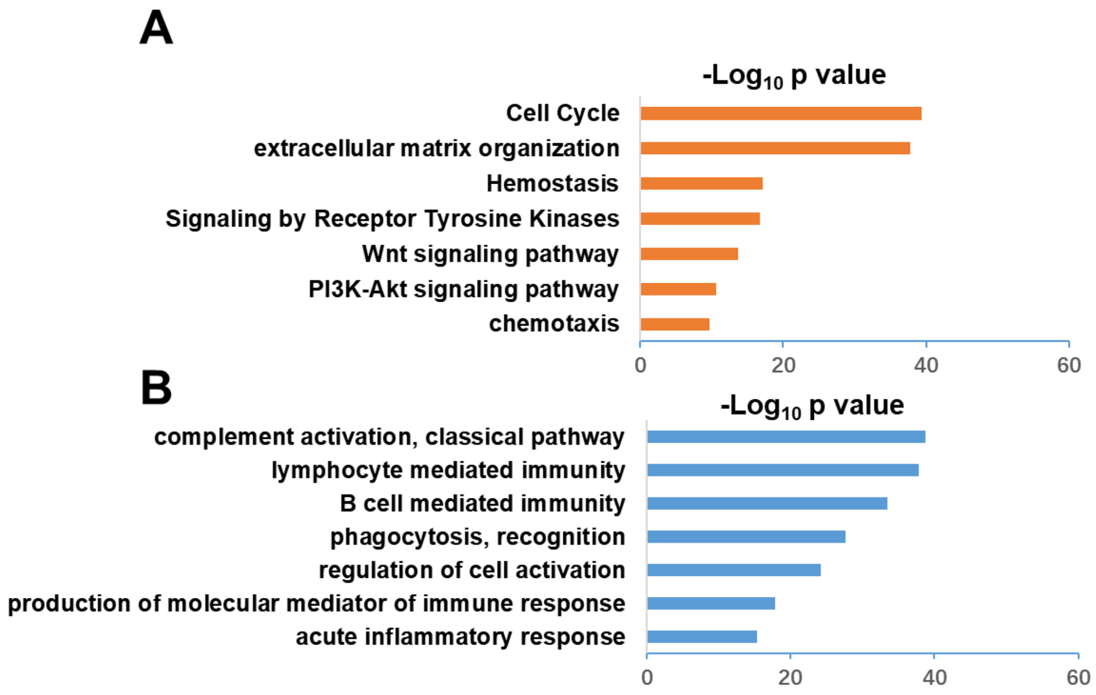

Figure S6. GO analysis of the DEGs of MSC-induced CD23$^+$CD43$^+$ B cells

GO and pathway enrichment analysis of the DEGs of MSC-induced CD23$^+$CD43$^+$ B cells. (A) Gene ontology (GO) and pathway enrichment analysis of upregulated genes by the metascape tool. (B) GO and pathway enrichment analysis of downregulated genes by the metascape tool.

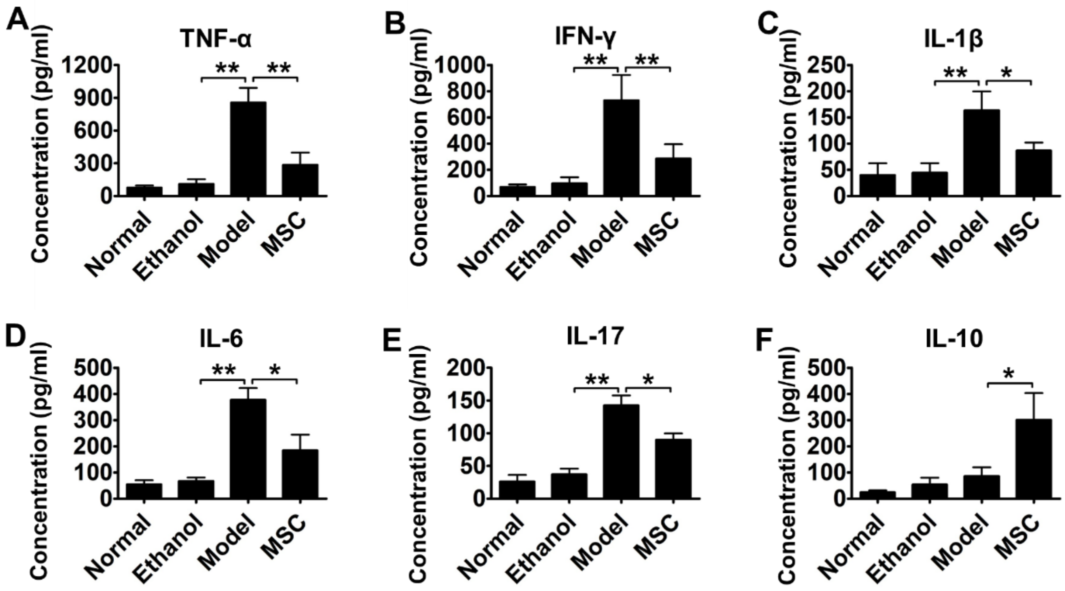

Figure S7. Alteration of serum inflammatory cytokines in colitis mice treated with hMSCs.

Serum TNF-α, IFN-γ, IL-1β, IL-6, IL17, and IL-10 in colitis mice treated with and without hMSC were detected by ELISA. Data are presented as the mean ± SD for each group. $*p < 0.05$, $**p < 0.01$.

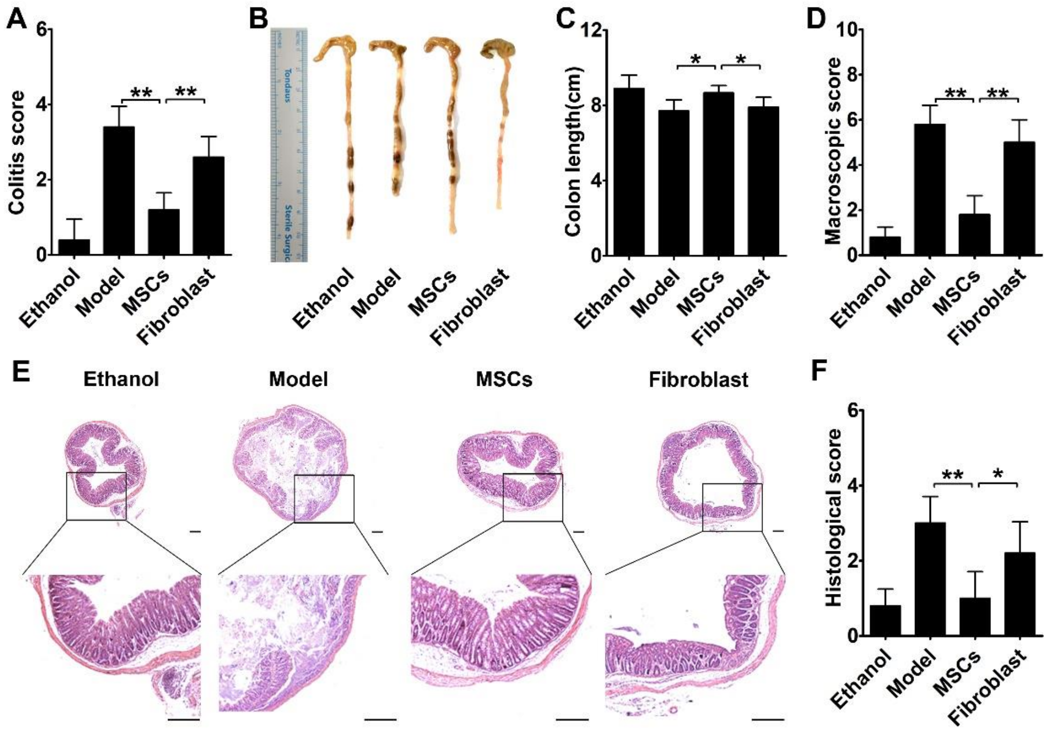

Figure S8. Fibroblasts could not alleviate colitis in mice.

Using the mice colitis, we evaluated the therapeutic effects both of hMSCs and human fibroblasts. (A) Colitis score of mice. (B) Representative colonic length of mice. (C) Quantification of the colonic length of mice. (D) Macroscopic damage score of colons. (E) Representative histological changes of colons. (F) Histological score of colons. Data represent mean values ± SD of five mice per group. *p<0.05; **p<0.01.

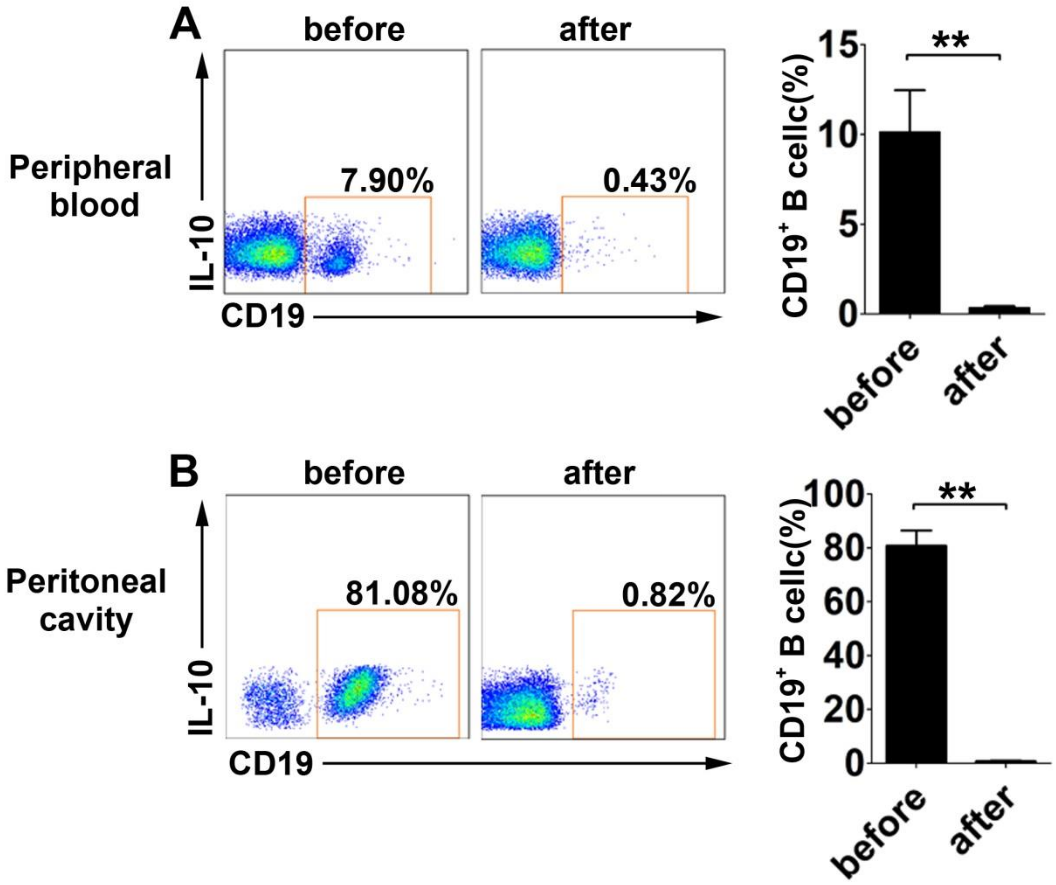

Figure S9. Alteration of B cells before and after anti-CD20 antibody administration.

Mice were injected i.v. with 250 μg of anti-mouse CD20 antibody. B cells were detected at day 7 after treatment. (A) Representative plot of B cells in peripheral blood after treatment. (B) Representative plot of B cells in the peritoneal cavity after treatment.
